# Demonstration of a novel technique to quantitatively assess inflammatory mediators and cells in rat knee joints

**DOI:** 10.1186/1476-9255-4-13

**Published:** 2007-06-13

**Authors:** Nicola J Barton, David A Stevens, Jane P Hughes, Adriano G Rossi, Iain P Chessell, Alison J Reeve, Daniel S McQueen

**Affiliations:** 1 Division of Neuroscience, University of Edinburgh, Medical College, 1 George Sq, Edinburgh, EH8 9JZ, UK; 2 Neurology CEDD, GlaxoSmithKline R&D Ltd, Harlow, Essex CM19 5AW, UK; 3 MRC Centre for Inflammation Research, The Queens Medical Research Institute, University of Edinburgh, EH16 4TJ, UK

## Abstract

**Background:**

The inflammation that accompanies the pain and swelling associated with osteo- and rheumatoid arthritis is mediated by complex interactions of inflammatory mediators. Cytokines play a pivotal role in orchestrating many of these processes, including inflammatory cell recruitment, adhesion and activation. In addition, prostaglandins are secreted into the synovial cavity and are involved in perpetuation of local inflammation, vasodilatation and vasoconstriction, and also with bone resorption. Pre-clinical models have been developed in order to correlate to the human disease and principle among these is the adjuvant-induced arthritis model in the rat.

**Methods:**

We have developed a technique to quantitatively assess the contents of synovial fluid samples from rat joints. Two needles joined together are inserted into the knee joint of anaesthetised rats and connected to a Watson-Marlow perfusion pump. Sterile saline is infused and withdrawn at 100 μl min^-1 ^until a 250 μl sample is collected.

**Results:**

Our results demonstrate up to 125 fold increases in synovial IL1α and IL1β concentrations, approximately 30 fold increases in levels of IL6 and IL10 and a 200–300 fold elevation in synovial concentrations of TNFα during FCA-induced experimental arthritis. Finally, this novel technique has demonstrated a dose-response relationship between FCA and the total cell counts of synovial perfusates.

**Conclusion:**

In summary, this new technique provides a robust method for quantifying inflammatory mediators and cells from the synovial cavity itself, thereby detailing the inflammatory processes from within the capsule and excluding those processes occurring in other tissues surrounding the entire articulation.

## 1. Background

Inflammatory joint diseases such as rheumatoid arthritis (RA) are regulated by complex interactions involving many mediators, such as prostanoids and cytokines. The infiltration of cells into the synovial tissue and joint space is another key characteristic of synovitis, which combined with release of these mediators and degradative enzymes, eventually leads to cartilage and bone destruction (for reviews see [1]).

Measuring the levels of these mediators of inflammation in the synovial fluid from patients can provide information about the underlying pathophysiology of joint disease [2], for example the level of severity and current activity [3-5] as well as inter-individual variations in disease [6] and effectiveness of drug-treatments (for review see [7]). Furthermore changes occurring in the synovial fluid can be used as biomarkers of disease; this has already been demonstrated in RA patients with plasma levels of inflammatory proteins [8,9].

Human joint fluid samples have been taken and analysed for inflammatory mediator content from both healthy volunteers and patients with joint diseases. These studies revealed the importance of particular cytokines, including Tumour Necrosis Factor (TNF)α, Interleukin (IL) 1β, and IL6, which are now targets for disease-modifying anti-rheumatic drugs (DMARDs; for review see [10,11]). Furthermore increases in virtually all the prostanoids have been detected from these samples [12,13], but notably Prostaglandin E_2 _(PGE_2_), which has been associated with erosion of bone and cartilage in RA [14-17].

Although studies have investigated the fluid taken from joints, most research has focused on the inflammatory mediators within the synovial membrane, rather than those released into the intra-articular space. One reason for this is the technical difficulty of trying to assess cytokine levels in such a viscous material as synovial fluid. Several studies have assessed cytokine gene expression levels in the synovial membrane, rather than the actual protein content, both in human clinical samples [18,19] and in animal models of arthritis [20-22]. In addition, PGE synthase, the enzyme responsible for the conversion of cyclooxygenase-derived PGH_2 _to PGE_2 _has been detected in synovial tissues of patients with RA [23].

The early time course of release of key mediators cannot be determined using human synovial fluid samples, as patients rarely report to the clinic until the disease has progressed and is causing chronic pain and swelling [24]. Even then, repeated sampling from individuals is difficult, and most patients are prescribed drugs, to improve their symptoms and quality of life, which interfere with inflammatory regulatory processes and cytokine expression. Therefore by using animal models of disease, the early events of inflammation can be elucidated, and the effects of drugs on inflammatory markers can be measured under controlled conditions.

Rat adjuvant-induced unilateral arthritis is a well established RA disease model. [25-27] and use of this model has gone a long way in aiding the understanding of the time-course of the pathology in clinical RA. The model closely mimics the pathology of human RA, including histopathological changes, cell infiltration, hypersensitivity and swelling of the affected joint [28-30]. Previous studies in animal models of joint inflammation have investigated the time course of cytokine protein or gene expression using homogenates of whole rat joints or paws *post mortem *[20-22,31-33]. A major limitation of these studies is that such sampling always includes bone, synovial tissue, synovial fluid and surrounding muscles and connective tissue, which will not allow the origin of any analytes to be determined. Others have surgically dissected and lavaged knee joints in order to collect the synovial fluid from dead animals [34-36]. However, this does not allow for acute repeated sampling from the same animal over a period of up to a day to determine the affect of drugs on the levels of inflammatory mediators, or the acute effect of an inflammatory insult on inflammatory processes in the synovial cavity, a significant benefit of the perfusion method described here. A further study used an *in vivo *microdialysis procedure to determine the levels of inflammatory mediators in the synovial fluid of rats with adjuvant induced polyarthritis [37]. However, the apparatus used for this had limitations, for example the molecular weight cut-off of the microdialysis membrane was 50 kD, and therefore potentially underestimated the levels of IL1β in the joints. Furthermore, this limits the molecules that could be assessed by this method, which is in contrast to the present method, in which there is no limit to the size of molecules collected. The perfusion technique described in the present study also allows for the collection of cells from the joint space. As yet, no studies appear to have been carried out by perfusing saline through the intact joint space and collecting samples of cells and mediators from intact anaesthetised animals. The primary aim of this study was to develop a perfusion method to sample only the synovial fluid. A secondary aim was to study the effects of a joint insult on the intra-articular cytokine concentrations and cell infiltrate levels associated with adjuvant-induced arthritis in the joint space were also measured, as these are known key mediators in human RA conditions.

## 2. Methods

Experiments were performed in accordance with Home Office regulations and within UK animal welfare guidelines, and received Local Ethics Committee approval. Male Wistar rats (Charles River, UK; initial weight ranges 240–290 g) were used. Rats were housed four to a cage in a 12-h light: dark environment and were given free access to standard animal feed and water for the duration of the study.

### 2.1 Arthritis induction

Briefly, rats (8) were transiently anaesthetised using 3% halothane in oxygen. The left knee was injected with 150 μl of Freund's Complete Adjuvant (FCA; 1 mg ml^-1 ^Mycobacterium tuberculosis, Sigma, UK; i.art). A further 3 rats received a higher dose of FCA (500 μg), in order to assess the effect of adjuvant dose on inflammatory cell recruitment and mediator release into the joint space (100 μl; 5 mg ml-1 Mycobacterium tuberculosis, MAFF, UK; i.art). Only 3 rats were used for this part of the study, as it was designed as a pilot study to determine whether differences in the number of inflammatory cells and mediators present in the knee joint were evident between normal animals and those injected with the two doses of adjuvant using this new technique. The right joints were untreated. Animals were then allowed to recover from the anaesthesia.

### 2.2 Perfusion of joint space and analysis of samples

#### 2.2.1. The perfusion needles

**Figure 1:**
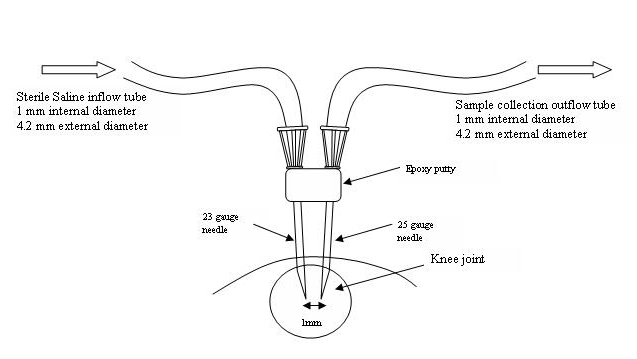
The perfusion needles and the perfusion system managing inflow and outflow from the knee joint space. A Watson-Marlow pump controlled the rate of saline infusion and sample extraction (100 μl min^-1^) from the joint. After the knee was secured to prevent movement of the limb, needles were inserted into the knee joint through the patella tendon.

A needle perfusion system was constructed by binding a 25- and a 23-gauge needle together using epoxy putty, with the bevels of the needles positioned on the outside edges facing away from one another (see Figure 1). The tips of the needles were set 1–1.5 mm apart.

#### 2.2.2. Perfusion of knee joints

Rats were anaesthetised with urethane (ethyl carbamate; 0.6 ml 100 g^-1 ^body weight; 25% w v^-1 ^solution; single i.p. injection). Once fully anaesthetised the animal was laid on its back on an automated heating blanket (Harvard Apparatus Limited, UK) and its core body temperature maintained at 37°C via a thermistor probe positioned in the rectum.

The limbs of the rat were flexed over a 20 ml glass vial, with the patella facing directly upwards for insertion of the perfusion needles, and the limb was secured in place with tape. The 23-gauge needle was connected to a Watson-Marlow roller pump via silicone rubber perfusion tubing (internal diameter 1 mm, external diameter 4.2 mm, Watson Marlow, UK). Sterile saline was infused at a constant rate of 100 μl min^-1^. After infusion of 100 μl of vehicle (sterile saline), the outflow tubing was connected to the 25-gauge needle, to minimise pressure build-up within the joint space. Fluid was infused and withdrawn at a constant rate until a 250 μl basal sample was collected in a 1.5 ml centrifuge tube. Samples were immediately frozen at -20°C.

#### 2.2.3 Cytokine assay of joint samples

##### Luminex assay

Samples from the studies investigating the effects of anaesthetic on joint cytokine levels (n = 10) and the differences between normal (n = 10), high dose FCA-injected (n = 3) and low dose FCA- injected joints (n = 8) were analysed using a multi-cytokine bead array detection system capable of detecting rat IL1α, IL1β, IL2, IL4, IL6, IL10, Interferon (IFN) γ, Granulocyte Macrophage-Colony Stimulating Factor (GM-CSF) and TNFα, according to the manufacturers instructions (Bio-Rad cytokine rat 9-plex, Biorad, USA). Briefly, a monoclonal antibody directed against the desired analyte was covalently coupled to dyed 5.5 μm polystyrene beads (2.5 × 10^6 ^beads ml^-1 ^cytokine^-1^). The conjugated beads were exposed to 50 μl of sample or standard solutions containing a known amount of cytokine, in a 96-well filter plate and incubated overnight at 4°C, protected from light. After a series of washes and vacuum filtration to remove unbound protein, a biotinylated detection antibody specific for a different epitope on the analyte was added to the reaction. After incubation, the unbound antibody was removed; the reaction mixture was detected by the addition of streptavidin-phycoerythrin (streptavidin-PE), which binds to the biotinylated detection antibodies. Following a further series of washes and vacuum filtration, the beads were re-suspended in 200 μl 5% BSA in PBS; the plate was stored at 4°C in the dark until analysis. The reaction mixture was read using a Luminex Data Collector in a Luminex 100 flow cytometer (Luminex, USA). The minimum detection limit of the assay was 2 pg ml^-1 ^for each mediator measured. Any values lower than these levels were classed as 0 for the purposes of this study.

##### Luminex data analysis

Excel data files were generated containing individual bead numbers and the associated median fluorescence intensities. Standard curves were plotted to calculate the relative amount of each cytokine in samples, using the aliquoted serial dilutions of a positive control solution for calibration. Unknown sample cytokine concentrations were calculated from the curve.

##### ELISA assay

The levels of TNFα and ILβ in samples from studies investigating the effects of the needles (n = 6), and leakage of infusion from the joint cavity (n = 2) were measured using commercially available ELISA kits that specifically recognize the rat cytokines (BioSource International, Camarillo, USA) according to the manufacturer's instructions. Briefly, 100 μl aliquots of sample were pipetted into the wells of a microtiter plate pre-coated with an antibody specific for rat IL-1β or TNFα and incubated for 3 h at room temperature. After washing, a different biotinylated anti-rat IL-1β or TNFα antibody was added and incubated at ambient temperature for 1 h. Streptavidin-peroxidase was added and incubated for 30 min. After a third incubation and washing to remove all unbound enzyme, colour was developed by addition of stabilized chromogen (tetramethylbenzidine), a stop solution added and the intensity of the coloured product quantified spectrophotometrically at 450 nm. The minimum detection limit of the assay was 2 pg ml^-1^.

### 2.3 Study design

#### 2.3.1 Anaesthetic effects

In order to determine what effect anaesthetic agents had on inflammatory mediators in joints, control experiments were carried out. Firstly, five naive rats were anaesthetised with urethane (ethyl carbamate; 0.6 ml 100 g^-1 ^body weight; 25% w v^-1 ^solution; single i.p. injection), and five further rats with sodium pentobarbital (1 ml kg^-1 ^body weight; 60 mg ml^-1 ^solution; single i.p. injection maintained with i.v. 375 μl hr^-1 ^20 mg ml^-1 ^solution of pentobarbital). No other procedures were carried out for 7 hours, at which point perfusion needles were inserted into both knee joints and a 250 μl sample collected. The sample was frozen immediately at -20°C, and later assayed using the Luminex assay.

#### 2.3.2 Needle effects

In order to determine what effects inserting the perfusion needles had on synovial cytokine concentrations, an experiment was carried out in which six animals were anaesthetised with urethane (as described above), and the perfusion needles inserted into both knee joints and held in position for 7 hours, at which time a 250 μl sample was collected. The sample was frozen immediately at -20°C, and later assayed using an ELISA.

#### 2.3.3 Perfusion effects on the concentration of analyte

Two naïve rats were anaesthetised with urethane (as described above) and a basal sample taken immediately. Then 1000 pg recombinant rat IL1β (Bioclone, USA) in 100 μl was infused over 1 min. A second sample was taken1 hour later; this was repeated hourly until 7 hours post-IL1β infusion. The samples were frozen and later assayed for IL1β content using an ELISA, to determine if the sample contained the same amount of IL1β that was initially infused.

#### 2.3.4 Cytokine levels in normal and FCA-injected joints

Basal samples from ipsilateral and contralateral joints of 10 normal animals were compared with basal samples from 8 rats which had received i.art low dose FCA (150 μg) and 3 that were injected with i.art high dose FCA (500 μg) 14 days earlier. Samples (250 μl) were collected and frozen for later testing with the Luminex bead array.

#### 2.3.5 Total cell counts

Joint perfusion samples were collected from ten naïve rat knee joints, eight 150 μg FCA-injected ipsilateral and contralateral joints and three 500 μg FCA-injected ipsilateral and contralateral joints. Undiluted samples were viewed by light microscopy in a haemocytometer. If red blood cells were present, or a high number of inflammatory cells, samples were diluted in saline, with added Zappoglobin, as per the manufacturer's instructions (1 drop per 20 ml).

### 2.4 Data Analysis

Data were collected and analysed using Microsoft Excel and Graphpad Prism software. Results are expressed as mean ± standard error of the mean (SEM) where appropriate.

#### Statistics

The Mann-Whitney U (non-parametric) test was used to analyse differences between groups, which were not normally distributed, or in which the sample size was small. To determine differences between the means of more than two groups a non-parametric one-way analysis of variance (Kruskal-Wallis) test was performed and a post-hoc test (Dunn's) undertaken if the test was significant. In all cases the null hypothesis was rejected at *P *< 0.05.

## 3. Results

### 3.1 Anaesthetic effects

Samples from naïve animals (n = 5) which received no treatment during 7 hours of urethane anaesthesia, showed a slight trend for increased levels of cytokines, but the increases were not statistically significant for IL1α, IL1β, IL2, IL4, IL6, IL10, GM-CSF, IFNγ, or TNFα compared with samples taken from rats immediately after administration of anaesthetic (n = 10; *P *> 0.05, Mann Whitney) see Table 1. However, in contrast, animals anaesthetised with pentobarbital (n = 5), had significantly higher levels of GM-CSF and TNFα (*P *< 0.05, Mann Whitney) after 7 hours, in comparison with naïve joints, see Table 1.

**Table 1:** The effect of anaesthetic on basal levels of cytokines in the joint.

	**IL1α**	**IL1β**	**IL2**	**IL4**	**IL6**	**IL10**	**GM-CSF**	**IFNγ**	**TNFα**
**Basal (n = 10)** Mean ± SEM	0.8 ± 0.5	1.0 ± 1.0	0.5 ± 0.5	0.2 ± 0.2	2.5 ± 2.5	1.5 ± 1.0	0 ± 0	0.1 ± 0.1	0.2 ± 0.2
**Urethane (n = 5)** Mean ± SEM	2.6 ± 1.1	0 ± 0	0 ± 0	0 ± 0	0 ± 0	0 ± 0	1.0 ± 0.6	0.3 ± 0.3	36.6 ± 20.9
**Pentobarbital (n = 5)** Mean ± SEM	1.4 ± 0.7	0.2 ± 0.2	0.1 ± 0.1	0 ± 0	0 ± 0	6.2 ± 6.2	1.7 ± 0.4*	0.1 ± 0.1	44.2 ± 21.6*

Levels of nine cytokines in rats anaesthetised for 7 hours with either urethane or pentobarbital, in comparison with samples taken immediately after urethane anaesthesia (basal). Mann Whitney test were performed to determine differences between each anaesthetic and basal levels. Pentobarbital anaesthesia resulted in a significant elevation of GM-CSF and TNFα levels; statistical significance P < 0.05 indicated by *.

### 3. 2 Needle effects

**Figure 2:**
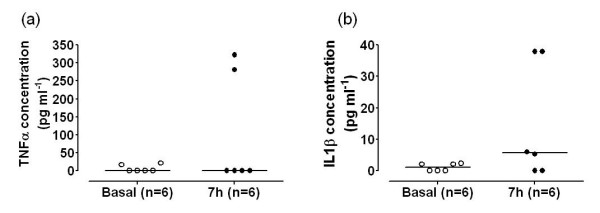
Levels of (a) TNFα and (b) IL1β from joints immediately after needle insertion (basal), and 7 hours later. Cytokines were assayed using an ELISA, and although there was an apparent increase in TNFα concentrations, to approximately 300 pg ml^-1 ^in two samples, this was not statistically significant (*P *> 0.05, Mann Whitney). The horizontal lines on the graphs represent the median values in each group.

Samples taken from knee joints in which the perfusion needles had been in place for 7 hours while the animal was anaesthetised with urethane (n = 6) showed increased levels of TNFα, as measured by ELISA, but these were not statistically significant from basal samples from the same rats immediately after needle insertion (*P *> 0.05, Mann Whitney). IL1β levels in two joints increased to approximately 40 pg ml^-1 ^over this time period, see Figure 2.

### 3.3 Perfusion effects on the concentration of analyte

Two joint perfusions were carried out to determine if any of the infused solution leaked from the joint space prior to withdrawal of samples. Recombinant rat IL1β (1000 pg), a cytokine known to be detectable by ELISA, was infused into the joint, along with saline, and samples were collected hourly. In both cases the full amount (1000 pg) administered was recovered in the first two samples. However, a greater amount of IL1β was recovered compared to the initial dose administered; Table 2 shows the results.

**Table 2:** Perfusion effects on the concentration of analyte.

	**Animal 1**		**Animal 2**	
	
	**IL1β concentration (pg ml^-1^; 250 μl)**	**Amount of IL1β (pg)**	**IL1β concentration (pg ml^-1^; 250 μl)**	**Amount of IL1β (pg)**
1 hour	2000	500	2000	500
2 hour	2000	500	2000	500
3 hour	200	50	356	89
4 hour	544	136	216	54
5 hour	350	87.5	210	52.5
6 hour	458	114.5	200	50
7 hour	318	79.5		

**Total (pg)**	**1467.5**		**1245.5**	

IL1β concentrations in each 250 μl sample collected, up to 7 hours post-infusion of IL1β (1000 pg). The amount of IL1β protein in each sample was calculated and summed, to show that little or no leakage from the joint space occurred. In fact, more IL1β was present than was injected, in both cases as a result of *de novo *release of endogenous IL1β protein.

### 3.4 Levels of cytokines in normal and FCA-injected joints

**Figure 3:**
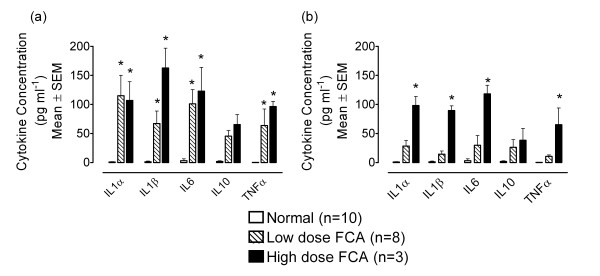
Levels of IL1α, IL1β, IL6, IL10 and TNFα in (a) ipsilateral and (b) contralateral joints of normal rats and those injected with low (150 μg; n = 8) and high (500 μg; n = 3) dose FCA 14 days earlier. There were negligible levels of any of the mediators measure in naïve joints (n = 10), but a significant increase in the expression of IL1α, IL1β, IL6 and TNFα was seen in all ipsilateral inflamed joints and in contralateral joints of rats injected with the high dose FCA (*P *< 0.05, Two-way ANOVA; compared with normal joints); statistical significance represented by *.

Fourteen days after rats received 150 μg or 500 μg FCA i.art (n = 8 and 3 respectively), the ipsilateral joint contained significantly higher levels of IL1α, IL1β, IL6 and TNFα compared with samples from naïve joints (n = 10), as measured by the Luminex assay (*P *< 0.05, Two-way ANOVA; see Figure 3a). The contralateral joints of rats injected with 500 μg FCA also contained significantly higher levels of IL1α, IL1β, IL6 and TNFα (*P *< 0.05, Two-way ANOVA; see Figure 3b).

### 3.5 Total cell counts

**Figure 4:**
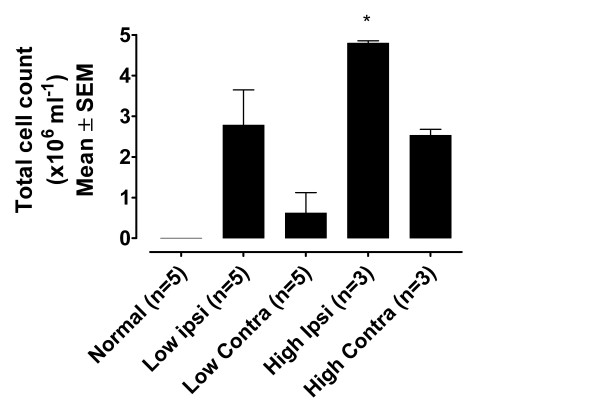
The effects of 150 μg (low dose; n = 5) and 500 μg (high dose; n = 3) FCA on total cell count from joint perfusates. Naïve joints contained no cells (0), whereas all other joints contained increased levels, although only high dose ipsilateral joints proved to have significantly raised levels (*P *< 0.05, Mann Whitney); statistical significance donated by *.

Total inflammatory cell counts from normal animals (n = 5) and those injected with FCA (n = 8) 14 days prior to sampling are shown in Figure 4. Normal joints had no cells detectable, whereas all others samples had measurable levels. However, only the 500 μg FCA ipsilateral (n = 3) joints proved to have a significantly greater number of cells than normal joints (4.8 ± 0.06 × 10^6 ^cells ml^-1^; P < 0.05, Mann Whitney). A dose-response relationship was demonstrated by the total cell count in both ipsilateral and contralateral joints.

## 4. Discussion

The main aim of this study was to develop a method for sampling synovial fluid from the knee joint of anaesthetized rats. The technique was firstly validated by assessing whether any inflammatory response was evoked by the experimental set up, including the anaesthetic or the needles themselves; the efficiency of the system was investigated, *i.e*. whether any infused solution leaked from the joint space prior to sample extraction. Once the above factors had been assessed, they were taken into consideration when comparing samples from naïve and adjuvant- injected inflamed joints. Finally, the novel perfusion technique was used to quantify inflammatory cell numbers within the rat synovial cavity. This technique proved to be reliable and consistent when perfusing the joint cavity, and regular volumes of sample were easily collected. There were no problems with measuring protein content due to high sample viscosity, and this technique is therefore a valuable addition to protocols which use homogenates of entire joints to assess inflammatory mediator content.

It was established that the choice of anaesthetic may play a role in initiating an inflammatory response within the knee joints. Urethane, a hypnotic anaesthetic agent commonly used for laboratory animals, resulted in very little change in any of the mediators measured over a 7 hour period. In contrast, pentobarbital (pentobarbitone), a short-acting anaesthetic which must be maintained by i.v infusion, therefore requiring further surgical preparation of the animal, induced increases in GM-CSF and TNFα after continuous administration during the day, perhaps a result of the surgery of the implanted cannulae. It was therefore decided to use urethane for experiments, given that it provides an extended period of anaesthesia with minimal physiological changes [38], without the need for invasive surgical preparation. Furthermore, pentobarbital can cause respiratory depression in rats, whereas urethane causes minimal cardiopulmonary disturbances [38,39].

Once it was established that urethane anaesthesia had no adverse effects on the system, it was necessary to evaluate any inflammatory component as a result of the perfusion needles themselves, over a sustained time period of 7 hours. It was noted that a few rats developed increased TNFα or IL1β levels as a result of the needles being maintained within the joint. However, the change occurred in only 20% of animals, and was not significant; moreover, the increases in the two mediators did not occur in the same animals.

This study has demonstrated that very little, if any, solution infused into the joint is lost into the surrounding tissue, and can be recovered in full through the effusion tubes. This was confirmed by injection of Evans blue dye into the joint cavity and later dissection of the tissue (data not shown here). Furthermore, there was an increased quantity of IL1β detected in the perfusate collected. Although this study was not designed to show the effects of the protein on the joint, the 1 ng dose of IL1β administered resulted in *de novo *release of natural IL1β, as shown by the fact that elevated levels of IL1 were detected, in addition to the 1 ng dose.

Adjuvant-induced arthritis is a widely used model of inflammatory joint disease, and will be the primary subject of future studies applying this novel perfusion method. It was therefore important that samples collected in this way could detect differences between cytokine levels in naïve joints and FCA-treated joints. Levels of all cytokines measured in this study (IL1α, IL1β, IL6, IL10 and TNFα) showed dramatic increases 14 days after an initial inflammatory insult to the joint, including high and low doses of FCA. Furthermore, the contralateral joint of rats injected with the high dose of FCA also had higher levels of all cytokines measured, illustrating the contralateral effect also noted in the inflammatory cell count study. Finally, this study investigated the total number of white blood cells present in the joint washout samples. Not surprisingly it was observed that FCA-injected joints contained higher levels than normal rat knee joints, as previously shown [40]. However, of particular interest are the cell counts in contralateral, non-injected limbs. Contralateral effects arising from a unilateral insult is a well documented phenomenon. In general, contralateral changes in behaviour, magnitude of biochemical fluctuations or histopathological lesions are less than those observed on the ipsilateral side (for review see [41]). Total cell count data from this study are in agreement with this finding, and although the lower dose of FCA used here does not elicit behavioural signs of inflammation or hypersensitivity in the contralateral joint, there is evidence of infiltration of inflammatory cells.

## 5. Conclusion

In summary, we have demonstrated the use of a novel method for sampling synovial fluid and washing out the joint cavity to collect the "inflammatory soup", and have performed assays to measure levels of cytokines during adjuvant-induced arthritis. This method has the advantage of enabling the contents of synovial fluid to be investigated alone, without the contamination of the surrounding tissue. We have also revealed its value in measuring cellular components of inflammation. In conclusion, as this new method of joint perfusion uses anaesthetised animals, acute effects of anti-inflammatory drugs or novel compounds could be investigated, thus improving the knowledge of how novel drug targets are affecting the inflammatory process.

## Competing interests

The author(s) declare that they have no competing interests.

## Authors' contributions

NJB planned and carried out all *in vivo *studies, *in vitro *assays, *data *interpretation, statistical analysis and compilation of the manuscript. DAS and JPH assisted with the Luminex assay use and data collection, then read and edited the manuscript after completion. AGR assisted with the total inflammatory cell count studies and reviewed and edited the article. IPC, AJR and DSM contributed intellectually to the experimental designs, as well as to structural and editorial aspects of the paper. All authors read and approved the final manuscript.

## References

[B1] Sweeney SE, Firestein GS (2004). Rheumatoid arthritis: regulation of synovial inflammation. Int J Biochem Cell Biol.

[B2] Kubota E, Kubota T, Matsumoto J, Shibata T, Murakami KI (1998). Synovial fluid cytokines and proteinases as markers of temporomandibular joint disease. J Oral Maxillofac Surg.

[B3] Alstergren P, Ernberg M, Kvarnstrom M, Kopp S (1998). Interleukin-1beta in synovial fluid from the arthritic temporomandibular joint and its relation to pain, mobility, and anterior open bite. J Oral Maxillofac Surg.

[B4] Chang H, Israel H (2005). Analysis of inflammatory mediators in temporomandibular joint synovial fluid lavage samples of symptomatic patients and asymptomatic controls. J Oral Maxillofac Surg.

[B5] Rooney M, Symons JA, Duff GW (1990). Interleukin 1 beta in synovial fluid is related to local disease activity in rheumatoid arthritis. Rheumatol Int.

[B6] Ulfgren AK, Grondal L, Lindblad S, Khademi M, Johnell O, Klareskog L, Andersson U (2000). Interindividual and intra-articular variation of proinflammatory cytokines in patients with rheumatoid arthritis: potential implications for treatment. Ann Rheum Dis.

[B7] Barrera P, Boerbooms AM, van de Putte LB, van der Meer JW (1996). Effects of antirheumatic agents on cytokines. Semin Arthritis Rheum.

[B8] Eastgate JA, Symons JA, Wood NC, Grinlinton FM, di Giovine FS, Duff GW (1988). Correlation of plasma interleukin 1 levels with disease activity in rheumatoid arthritis.

[B9] Houssiau FA, Devogelaer JP, Van Damme J, de Deuxchaisnes CN, Van Snick J (1988). Interleukin-6 in synovial fluid and serum of patients with rheumatoid arthritis and other inflammatory arthritides. Arthritis Rheum.

[B10] Christodoulou C, Choy EH (2006). Joint inflammation and cytokine inhibition in rheumatoid arthritis. Clin Exp Med.

[B11] Zwerina J, Redlich K, Schett G, Smolen JS (2005). Pathogenesis of rheumatoid arthritis: targeting cytokines. Ann N Y Acad Sci.

[B12] Egg D (1984). Concentrations of prostaglandins D2, E2, F2 alpha, 6-keto-F1 alpha and thromboxane B2 in synovial fluid from patients with inflammatory joint disorders and osteoarthritis. Z Rheumatol.

[B13] Trang LE, Granstrom E, Lovgren O (1977). Levels of prostaglandins F2 alpha and E2 and thromboxane B2 in joint fluid in rheumatoid arthritis. Scand J Rheumatol.

[B14] Dayer JM, Krane SM, Russell RG, Robinson DR (1976). Production of collagenase and prostaglandins by isolated adherent rheumatoid synovial cells. Proc Natl Acad Sci U S A.

[B15] Fulkerson JP, Damiano P (1983). Effect of prostaglandin E2 on adult pig articular cartilage slices in culture. Clin Orthop Relat Res.

[B16] Robinson DR, Smith H, McGuire MB, Levine L (1975). Prostaglandin synthesis by rheumatoid synovium and its stimulation by colchicine. Prostaglandins.

[B17] Robinson DR, Tashjian AH, Levine L (1975). Prostaglandin-stimulated bone resorption by rheumatoid synovia. A possible mechanism for bone destruction in rheumatoid arthritis. J Clin Invest.

[B18] Firestein GS, Alvaro-Gracia JM, Maki R (1990). Quantitative analysis of cytokine gene expression in rheumatoid arthritis. J Immunol.

[B19] Wagner S, Fritz P, Einsele H, Sell S, Saal JG (1997). Evaluation of synovial cytokine patterns in rheumatoid arthritis and osteoarthritis by quantitative reverse transcription polymerase chain reaction. Rheumatol Int.

[B20] Patten C, Bush K, Rioja I, Morgan R, Wooley P, Trill J, Life P (2004). Characterization of pristane-induced arthritis, a murine model of chronic disease: response to antirheumatic agents, expression of joint cytokines, and immunopathology. Arthritis Rheum.

[B21] Rioja I, Bush KA, Buckton JB, Dickson MC, Life PF (2004). Joint cytokine quantification in two rodent arthritis models: kinetics of expression, correlation of mRNA and protein levels and response to prednisolone treatment. Clin Exp Immunol.

[B22] Thornton S, Duwel LE, Boivin GP, Ma Y, Hirsch R (1999). Association of the course of collagen-induced arthritis with distinct patterns of cytokine and chemokine messenger RNA expression. Arthritis Rheum.

[B23] Westman M, Korotkova M, af Klint E, Stark A, Audoly LP, Klareskog L, Ulfgren AK, Jakobsson PJ (2004). Expression of microsomal prostaglandin E synthase 1 in rheumatoid arthritis synovium. Arthritis Rheum.

[B24] Cohen SB, Katsikis PD, Chu CQ, Thomssen H, Webb LM, Maini RN, Londei M, Feldmann M (1995). High level of interleukin-10 production by the activated T cell population within the rheumatoid synovial membrane. Arthritis Rheum.

[B25] Bileviciute I, Lundeberg T, Ekblom A, Theodorsson E (1993). Bilateral changes of substance P-, neurokinin A-, calcitonin gene-related peptide- and neuropeptide Y-like immunoreactivity in rat knee joint synovial fluid during acute monoarthritis. Neurosci Lett.

[B26] Billingham ME, Pettipher ER (1995). Mechanisms and Models of Rheumatoid Arthritis.

[B27] Mapp PI, Terenghi G, Walsh DA, Chen ST, Cruwys SC, Garrett N, Kidd BL, Polak JM, Blake DR (1993). Monoarthritis in the rat knee induces bilateral and time-dependent changes in substance P and calcitonin gene-related peptide immunoreactivity in the spinal cord. Neuroscience.

[B28] Donaldson LF, Seckl JR, McQueen DS (1993). A discrete adjuvant-induced monoarthritis in the rat: effects of adjuvant dose. J Neurosci Methods.

[B29] Pelegri C, Franch A, Castellote C, Castell M (1995). Immunohistochemical changes in synovial tissue during the course of adjuvant arthritis. J Rheumatol.

[B30] Wilson AW, Medhurst SJ, Dixon CI, Bontoft NC, Winyard LA, Brackenborough KT, Alba JD, Clarke CJ, Gunthorpe MJ, Hicks GA (2006). An animal model of chronic inflammatory pain: Pharmacological and temporal differentiation from acute models. European Journal of Pain.

[B31] Magari K, Miyata S, Nishigaki F, Ohkubo Y, Mutoh S, Goto T (2003). Differential effects of FK506 and methotrexate on inflammatory cytokine levels in rat adjuvant-induced arthritis. J Rheumatol.

[B32] Marinova-Mutafchieva L, Williams RO, Mason LJ, Mauri C, Feldmann M, Maini RN (1997). Dynamics of proinflammatory cytokine expression in the joints of mice with collagen-induced arthritis (CIA). Clin Exp Immunol.

[B33] Smith-Oliver T, Noel LS, Stimpson SS, Yarnall DP, Connolly KM (1993). Elevated levels of TNF in the joints of adjuvant arthritic rats. Cytokine.

[B34] Keeble JE (2004). The role of sensory nerves in joint inflammation: studies using TRPV1 knockout mice. pA2 online.

[B35] Singh HN, Blancuzzi V, Greenwood S, Skiles JW, O'Byrne EM (1997). Synovial fluid levels of tumor necrosis factor-alpha in the inflamed rat knee: modulation by dexamethasone and inhibitors of matrix metalloproteinase and phosphodiesterase. Inflamm Res.

[B36] Vale ML, Benevides VM, Sachs D, Brito GA, da Rocha FA, Poole S, Ferreira SH, Cunha FQ, Ribeiro RA (2004). Antihyperalgesic effect of pentoxifylline on experimental inflammatory pain. Br J Pharmacol.

[B37] Liu SH, Wong CS, Chang DM (2005). Increase Monocyte chemoattractant protein-1 in knee joints of rats with adjuvant-induced arthritis: in vivo microdialysis. The journal of rheumatology.

[B38] Sapru HN, Krieger AJ (1979). Cardiovascular and respiratory effects of some anesthetics in the decerebrate rat. Eur J Pharmacol.

[B39] Wixson SK, White WJ, Hughes HC, Lang CM, Marshall WK (1987). The effects of pentobarbital, fentanyl-droperidol, ketamine-xylazine and ketamine-diazepam on arterial blood pH, blood gases, mean arterial blood pressure and heart rate in adult male rats. Lab Anim Sci.

[B40] Santos L, Tipping PG (1994). Attenuation of adjuvant arthritis in rats by treatment with oxygen radical scavengers. Immunol Cell Biol.

[B41] Shenker N, Haigh R, Roberts E, Mapp P, Harris N, Blake D (2003). A review of contralateral responses to a unilateral inflammatory lesion. Rheumatology (Oxford).

